# Supplementary material for: Patterns of Intron Gain and Loss in Fungi
Source: PLoS Biol. 2004 Nov 30;2(12):e422. doi: 10.1371/journal.pbio.0020422 (PMC532390; doi:10.1371/journal.pbio.0020422)
Supplement: Table S1 — Also available at http://genes.mit.edu/NielsenEtAl/. (4.3 MB ZIP). [file pbio.0020422.st001.zip › NielsenEtAl/html/733.html]

AN3035.1.NCU06089.1.MG00841.1.FG07043.1


```
 CLUSTAL W (1.82) Multiple Sequence Alignments - Introns Inserted


Sequence 1: MG00841.1	780 aa
Sequence 2: FG07043.1	747 aa
Sequence 3: NCU06089.1	758 aa
Sequence 4: AN3035.1	761 aa
Alignment Length: 822 aa
Number Identitical Residues: 195 aa
Alignment Score (without introns) 10862


MG00841.1 	MGIVG~LLPLLKSIQRPTELKKFAGETLAVDAYGWLHRGAVSCALELAQGKPTRK2YVDF
NCU06089.1	MGING~LLPLLKSIHRPAELRKYAGETLGIDGYGWLHRGAIACAIDLAQGKPTRK2YVDY
FG07043.1 	MGVSG1LLPLLKSIQKPTELKKYHGQTFGVDAYGWLHRAAYCCALELGQDKPTQK2YLNA
AN3035.1  	MGIKG1LHGLLKSIQKPCHLKKFSGQTLGVDAYGWLHRGTVACAVDLVLDRPTTK2HIDF
          	**: * *  *****::* .*:*: *:*:.:*.******.: .**::*  .:** * ::: 

MG00841.1 	AMHRVRMVKYFGATPYIVFDGDFLPSKALTEASRAKRREESKKAGMELLKAGKPAQAYQE
NCU06089.1	AMHRVKMFKHFGVTPYVVFDGDYLPSKAKTELDRESRREASRKTGLELLKAGKPSQAHIE
FG07043.1 	AMTRVRMLRHFGITPYMVFDGDFLPSKAATEDSRAKKRDEKKKAAMELLRAGKPAQATQE
AN3035.1  	VLSRVRMLLFFGVKPYLVFDGDNLPSKSGTELDRQQRRHESKTLGMELYRKGRTAEAYQE
          	.: **:*. .** .**:***** ****: ** .* .:*. .:. .:** : *:.::*  *

MG00841.1 	FQKAIDVTPEMARELIEELKKIDVPYVVAPYEADAQMVYLERNGYVSGIISEDSDLLVFG
NCU06089.1	LQKAIDITPEMARHLIDELKKANVPYVVAPYEADAQLIYLERRGIISGIVSEDSDLLVFG
FG07043.1 	FQKCIDITPEMASALIQLLKKLDIPYVVAPYEADAQLVYLERQGLINGIISDDSDLLVFG
AN3035.1  	LQKAVDVTPLMARQLIEELKKLDIQYVVAPYEADAQLVYLEKHGFIDGIISEDSDLLVFG
          	:**.:*:** **  **: *** :: ***********::***:.* :.**:*:********

MG00841.1 	AKRLLTKLDQHGQCIEVNRREFCAVREISLTGWTDAEFRQMAIFSGCDYLDGINKMGLKT
NCU06089.1	AKRLLTKMDQYGQCIEINRSQFCAVREISLTGWSDAEFRQMAIFSGCDYLDSLPSMGLRT
FG07043.1 	AKKLLTKLDQYGNCLEINRKDFCACREVSLTGWSDTEFRRMAIFSGCDYLDGLPGVGLKT
AN3035.1  	AKRLLSKLDQHGELIEINRADFTACREVSFVGWTDADFRRMCIMSGCDYLPNIARVGLKT
          	**:**:*:**:*: :*:** :* * **:*:.**:*::**:*.*:****** .:  :**:*

MG00841.1 	AYRMIRKHKTPEKVIRQLQFEGKHKISENYLAAFKQAELTFMHQRVFCPEKQELVLLTEP
NCU06089.1	AYRMIRKLKTPERIVKKLQFDGKIRVPADYLARFKQAELTFIYQRVFCPEKQAVVCLTEP
FG07043.1 	AYRMLRKTKVPERIVRMVQFQGK-RVSENYLTQFYQAELTFLHQWVFCPTKRELVHLTDL
AN3035.1  	AYRSIRKYKSVEKALRMLQFEGPYHVPADYLQSFMQAELTFLYQRVFCPKSGKLVPLTSP
          	*** :** *  *: :: :**:*  ::. :**  * ******::* **** .  :* **. 

MG00841.1 	TSGLDVEEMPFIGAKVDPELSRAIACGDVNPITKERIILP--------TPPSTKWRSFPT
NCU06089.1	DKSINVDDMPYIGAPIDAKLARAIAVGDVNPITKEPIIVT-------ATSPSKRRISQVF
FG07043.1 	DGTRTAEEMPFIGAFVEPEAARAIARGDMNPITKSMIISAP------TTTPSKRRHSQLA
AN3035.1  	DDGVNLDELPFIGADMDPETAVGVANGDLDPTSKKPLQLVIKPLGSSTVEQNKHIASLSR
          	      :::*:*** ::.: : .:* **::* :*. :    .. .:::.  ..:  *   

MG00841.1 	KSTVP-APSKPLGKPIEEFFKGHRRIPMGEMDANCFSVDP--QQVGALTANGLAPRVFPL
NCU06089.1	TSAFG-EGK-KMGKPIDQYFK-DRRIPLGEMDPNCFNVEP--DNGEQEATAEPRPIVFPL
FG07043.1 	TGAVD-QPP-PATKPISSYFRGHSRIPMGEMDPNCFEVDP--QRVLQITGGGLVPRVFPL
AN3035.1  	RQTIGSFSDIKPSKPINSFFT-PKRVPLAELDPNSLTPSPSQQRLLHRHVNSSWEPSLAP
          	  :. :       ***..:*    *:*:.*:*.*.:  .*:.:.             :. 

MG00841.1 	PRPYLEETIN-------RP--YTSSAAPRINRRRTEPIGNLLALSSSDQANRRRTSGP-I
NCU06089.1	PRPYVEDVGT--------SAAPARRYTAQNSRRKSEPISKLLASFDDTSSLTSRRQTT--
FG07043.1 	PRPYLDETTRPLPSNSRRPEANSTHTSPKLHRRRTEPVSKLLNSFTDTTATTRRTSSVGI
AN3035.1  	SRPSVARSTT----------VNDSSSRLSSPLVRSAERSLFLARTSKLTTLQPSKRQRLC
          	.** :                            ::   . :*    .  :          

MG00841.1 	RSSKDESRTAGFSTSPRPQKRARLCSEQHQSTKEDKELFAEKSKFFSTITKTPRSKRR--
NCU06089.1	GAAFEVFTDTDPAISTRPPKKARLCEDAVSED--AVTANPEKSKFFPQTKAKKAAARK--
FG07043.1 	KSSSSAVPSS--DPANRPPKKARLCDDNETGP--DEALPPQKSKFFP-VSKPRRSPRRTK
AN3035.1  	SETDEVIAAS------TPDCRSRFFAASSNDE------TPSGGQKLNRSKKARKSTLD--
          	  : .    :       *  ::*:               .. .: :   .    :     

MG00841.1 	SEGHIMSDDSIDDALLSLPDYGSLSSTHSSQPRKS--MDVFMDAR------SDDVTHETP
NCU06089.1	SESFIMSDDSIEEAFRSVPDAVWSSS--KARRRSG--KVQFVEESPSSEPVSQEKEQEDL
FG07043.1 	SDAYLLSDDSLDEALMALPDFDGWKP--SDKIKRG--VSVFVEKD------SQETSTTTV
AN3035.1  	----VFSDDATEDIMSQMPDPSEAANMTNEKFSAAGHSGESSEREDEVTKTNVPITTRSE
          	    ::***: :: :  :**        . :   ..      :   .    .        

MG00841.1 	D-VILATPMKD---DDQNKDTVEIEVPASSPIRESVSIEADAELDLAHQARRSSAKLGAA
NCU06089.1	EEPSLPAMPRSTTIDASSDAEDEVEVPGSSS-QEAITKKS----------VKSQTPLGPR
FG07043.1 	ESGSQLSFMKD---DETPLSSFDDSMPPPS---STVSLP-----------RTSSTPARPD
AN3035.1  	TEATQLDAKKQAYEAAFDSRTLSRKISAASEPQVVPQVT-----------DHHIKRQTST
          	 .       :.:          . .:. .*                            . 

MG00841.1 	LGRFAYTPGVTNSGNGTKMAYGLPTPESSGVPAAVAPRSTAEKKRDPSGSMETPRLTPLQ
NCU06089.1	LRQFSYQPGKPGARLVN----GLPTPSSTQERSAAVRRSSLN--------PSSPMLTPLQ
FG07043.1 	IRKFSYSKPTETPAS--------TTSQRSSIFSAASTPSTAP-------STAASRMTPLQ
AN3035.1  	LSKYSFKTDASGTRPNPQ-HSAVNGPERSASSQLVRQRTWTP--------TQPKRLTPLQ
          	: ::::      .    .   .   .. :     .   :             .  :****

MG00841.1 	RIHASAMQRHSPSASQGTNGTQTQKRRGPRRSLPPTAPVNPSFVPLPNVDLAEVAALSRG
NCU06089.1	RMGARALKQVTPRATPPS--SLRARKSSKPSEALGSVQVDPAAIPLPLADFAEVRALNMS
FG07043.1 	RLGARAMN--APMSPK-----LPKPRNSLDRQFLSGVPVNPAFVPLPKVNLDEVADLNKC
AN3035.1  	RLGQNALYRSRSLNNMP----ASPTISRSPSRIEDRTSVSSGDCPP--------AITLRT
          	*:   *:    .                        . *...  *               

MG00841.1 	GGGSEDLLVPNSDGENE-----PESPKVATSRTMDLARFLCR--
NCU06089.1	LQGSEDQIIPESDGETE-----LGTAATRP--RLNLAQYLYS--
FG07043.1 	G--SEDQIIPDSDEEDE-----DDEIDLPP-KKLNLSRFAFA--
AN3035.1  	GQGSEDLIVPDSEEEEDDCDAGDDSVSQSRVTALDLKRFSFTAR
          	  .*** ::*:*: * :...:.           ::* ::   :
```
